# Is Gemcitabine and Cisplatin Induction Chemotherapy Superior in Locoregionally Advanced Nasopharyngeal Carcinoma?

**DOI:** 10.12669/pjms.314.7374

**Published:** 2015

**Authors:** Wei Zheng, Sufang Qiu, Lingling Huang, Jianji Pan

**Affiliations:** 1 Wei Zheng, Department of Radiation Oncology, Cancer Hospital of Fujian Medical University, Fuzhou, Fujian, China. Fujian Provincial Key Laboratory of Translational Cancer Medicine, Fuzhou, Fujian, China. Teaching Hospital of Fujian Health College, Fujian Provincial Cancer Hospital, Fuzhou, Fujian, China; 2 Sufang Qiu, Department of Radiation Oncology, Cancer Hospital of Fujian Medical University, Fuzhou, Fujian, China. Fujian Provincial Key Laboratory of Translational Cancer Medicine, Fuzhou, Fujian, China. Teaching Hospital of Fujian Health College, Fujian Provincial Cancer Hospital, Fuzhou, Fujian, China; 3 Lingling Huang, Department of Radiation Oncology, Cancer Hospital of Fujian Medical University, Fuzhou, Fujian, China. Fujian Provincial Key Laboratory of Translational Cancer Medicine, Fuzhou, Fujian, China; 4 Jianji Pan, Department of Radiation Oncology, Cancer Hospital of Fujian Medical University, Fuzhou, Fujian, China. Fujian Provincial Key Laboratory of Translational Cancer Medicine, Fuzhou, Fujian, China. Teaching Hospital of Fujian Health College, Fujian Provincial Cancer Hospital, Fuzhou, Fujian, China

**Keywords:** Nasopharyngeal carcinoma, Radiotherapy, Induction chemotherapy

## Abstract

**Objective:**

To investigate the outcome of locoregionally advanced nasopharyngeal carcinoma (NPC) treated with induction chemotherapy followed by chemoradiotherapy.

**Methods:**

Between June 2005 and October 2007, 604 patients with locoregionally advanced NPC were analyzed, of whom 399 and 205 were treated with conventional radiotherapy and intensity-modulated radiotherapy (IMRT) respectively. Meanwhile, 153 patients received concurrent chemotherapy, and 520 were given induction chemotherapy.

**Results:**

With a median follow-up time of 65 months, the 3-, and 5-year overall survival (OS), locoregional free survival (LRFS), and distant-metastasis free survival (DMFS) rates were 82.5% vs. 72.6%, 90.6% vs. 87.1%, and 82.5% vs. 81.2%, respectively. Induction chemotherapy was not an independent prognostic factor for OS (P=0.193) or LRFS, but there was a positive tendency for DMFS (P=0.088). GP regimen (gemcitabine + cisplatin) was an independent prognostic factor for OS (P = 0.038) and it had a trend toward improved DMFS (P = 0.109). TP regimen (taxol + cisplatin) was only a significant prognostic factor for DMFS (P =0.038).

**Conclusions:**

Adding induction chemotherapy had no survival benefit, but GP regimen benefited overall survival and had a trend toward improved DMFS. GP regimen may be superior to TP/FP regimen (fluorouracil + cisplatin) in treating locoregionally advanced NPC.

## INTRODUCTION

Nasopharyngeal carcinoma (NPC) is a considered endemic carcinoma in Southern China, and radiotherapy (RT) is the main treatment modality for NPC.^1^ The 5-year overall survival (OS) rate ranged from 84-90% for early stage NPC patients, whereas locally advanced NPC patients had a poor 5-year OS rate with only 30.3-73.6%.^2^ Locoregional failure and distant-metastasis were the major patterns of treatment failure. With the prevailing use of IMRT, the 5-year OS and locoregional free survival (LRFS) rates have been up to 80% and 95%, respectively. However, the 5-year distant-metastasis free survival (DMFS) rate still reached to 77%.^3^ Although the IMRT is the most efficient treatment, distant metastasis is still the most common treatment failure pattern.

Concurrent chemoradiotherapy with or without adjuvant chemotherapy is the formerly standard care for locoregionally advanced nasopharyngeal carcinoma.^4^,^5^ Some randomized studies have confirmed the survival benefits through adding chemotherapy to conventional radiotherapy.^6^,^7^ In the intergroup 0099 trial,^4^ grade 3 and 4 toxicities in patients treated with concurrent chemoradiation therapy nearly doubled those who received irradiation only, 37% patients discontinued concurrent chemoradiation therapy prematurely due to the intolerance to combined treatment. However, the effects of adding chemotherapy have not been confirmed in IMRT era. Lin et al.^8^ demonstrated that improved local and/or regional control was the underlying factor of the improved survival for patients treated with concurrent chemoradiotherapy under IMRT. Whatever with conventional radiotherapy or IMRT, adjuvant chemotherapy has been confirmed as no survival benefit.^9^,^10^

Therefore, it is reasonable to question whether induction chemotherapy is able to offer benefits to OS by decreasing the rate of distant-metastasis or not. Although some studies have showed induction chemotherapy offers benefit for survival,^11^-^13^ other results were relatively disappointing.^14^,^15^ Under such circumstances, the efficacy of induction chemotherapy remains controversial. In addition, the most frequently used cisplatin-based induction chemotherapies in clinical practice include: TP regimen: Taxol + cisplatin, GP regimen: Gemcitabine + cisplatin, FP regimen: Fluorouracil + cisplatin.^16^-^18^ However, which protocol is more benefit for survival remains unclear.

Hence, we aimed to address the treatment outcomes and to analyze the effects of different induction chemotherapy regimens (TP regimen; GP regimen; FP regimen) in 604 NPC patients with locoregionally advanced disease, using Kaplan-Meier method and Cox proportional hazard model.

## METHODS

***Patients and pretreatment evaluation:*** Between June 2005 and October 2007, 816 patients with consecutive, newly diagnosed, pathologically proven NPC without distant metastases in our hospital were retrospectively included in the study. Patient participant approval was obtained from Fujian Provincial Cancer Hospital. Pretreatment evaluation consisted of a complete history and physical examination, flexible fiberoptic nasopharyngoscopy, complete blood counts, blood chemistries, chest X-ray or CT scans of the chest, electrocardiogram, abdominal ultrasonography, computed tomography (CT) scans of the nasopharynx and neck, bone emission computed tomography (ECT) scans, and dental evaluation. Magnetic resonance imaging (MRI) scans of the abdominal Ultrasonography and neck were performed instead of CT in all patients diagnosed after July 2005. Other tests and studies such as position emission tomography (PET) were performed at the treating physician’s discretion.

All cases were restaged according to the American Joint Cancer Committee 2010 staging classification. Patients who had evidence of distant metastasis were excluded from this analysis. Two hundred twelve patients who had early stage (Stage I and Stage II) disease were not eligible for this treatment protocol. Characteristics of patients with stage III to IVA NPC are listed in Table-I.

**Table-I:** Baseline characteristics of cohort.

Characteristic	n	%
*Gender*		
Male	466	77.15%
Female	138	22.85%
*Age(year)*		
≤50	395	65.40%
>50	209	34.60%
*Histology*		
WHOII+WHOIII	575	95.20%
WHOI	29	4.80%
*T classification*		
1	48	7.95%
2	61	10.10%
3	342	56.62%
4	153	25.33%
*N classification*		
0	49	8.11%
1	248	41.06%
2	247	40.89%
3	60	9.93%
*Stage*		
3	401	66.39%
4	203	33.61%
*Induction chemotherapy*		
No	84	13.91%
taxol + platinum (TP)	444	73.51%
gemcitabine + platinum (GP)	13	2.15%
fluorouracil + platinum (FP)	63	10.43%

### Radiotherapy

All patients received definitive radiotherapy. Among these patients, 399 (66.06%) patients were treated with conventional radiotherapy, and intensity-modulated radiotherapy (IMRT) was used in the remaining 205 (33.94%) patients. The detailed description of each of techniques used at Cancer Hospital of Fujian Medical University had been described previously.^19^,^20^ Salvage treatments (including intracavitary brachytherapy, IMRT, 3D-CRT, surgery, and chemotherapy) were provided for patients who developed relapse or persistent disease.

### Chemotherapy

Of the 604 patients who were given platinum-based chemotherapy, 153 (25.3%) received concurrent chemotherapy and 520 (86.09%) received induction chemotherapy. The induction chemotherapy consisted of 2 cycles of regimen as following: TP regimen: taxol (135 mg/m^2^ IV on the first day) + cisplatin (80 mg/m^2^ IV in days 1-3), GP regimen: gemcitabine (1000 mg/m^2^ IV in days 1,8)+ cisplatin (80 mg/m^2^ IV in days 1-3) and FP regimen: fluorouracil (800 mg/m^2^ IV in d1-d5) + cisplatin (80 mg/m^2^ IV in days 1-3). Induction chemotherapy spaced 2 weeks apart prior to the initiation of radiotherapy, and radiotherapy started within one week after the second cycle of chemotherapy. Concurrent chemotherapy consisted of cisplatin (80-100 mg/m^2^ given over day 1-3 of each 21-day cycle), taxol (135 mg/m^2^ IV on the first day) + cisplatin (80 mg/m^2^ IV in days 1-3), gemcitabine (1000 mg/m^2^ IV in days 1,8)+ cisplatin (80 mg/m^2^ IV in days 1-3) and fluorouracil (800 mg/m^2^ IV in d1-d5) + cisplatin (80 mg/m^2^ IV in days 1-3) at the discretion of the attending radiation oncologists. In addition, adjuvant cisplatin-based chemotherapy was given to 98 patients at the discretion of the attending radiation oncologists.

### Follow-up

The follow-up duration was calculated from the first day of diagnosis of NPC until death or the last follow-up. The median follow-up time was 65 months (range 3 to 86 months). All patients were evaluated weekly during treatment, and were required to be followed-up by their attending radiation oncologist after the completion of their treatment every three months in the first two years, every six months for three additional years, and annually thereafter. Each follow-up included a complete examination, flexible fiberoptic nasopharyngoscopy, basic serum chemistry, complete blood counts, chest X-ray or CT scans of the chest, and ultrasound of abdomen. Flexible fiberoptic endoscopy was performed at every visit after treatment. MRI of the head and neck areas was performed every 6 months.

### Statistics

OS duration was calculated from the start of diagnosis of NPC to the date of death or the date of the last follow-up visit. The duration of time to LRFS and DMFS was measured from the date of the completion of radiation therapy (including boost irradiation) until documented treatment failure. The survival rates were estimated by the Kaplan-Meier, and the statistical significance of differences were analyzed by the log-rank test. Cox proportional hazard model was performed for the aforementioned endpoints to define independent predictors among various potential prognostic factors. The level of statistical significance was set at a 2-tailed P-value of <0.05.

## RESULTS

### Treatment outcomes

In total, 93.21% of patients had complete follow-up. In the last follow-up, 73 patients (12.09%) developed disease relapse, and 115 (19.04%) had developed distant metastasis. Ultimately, 179 (29.64%) patients died at the end of this follow-up: 105 patients died from distant metastasis, 41 died from disease recurrence, 10 died from treatment complications, 15 died from other medical conditions, and 8 died from unknown reasons. The 3- and 5-year OS, LRFS, and DMFS rates were 82.5% vs. 72.6%, 90.6% vs. 87.1%, and 82.5% vs. 81.2%, respectively.

**Fig. 1:**
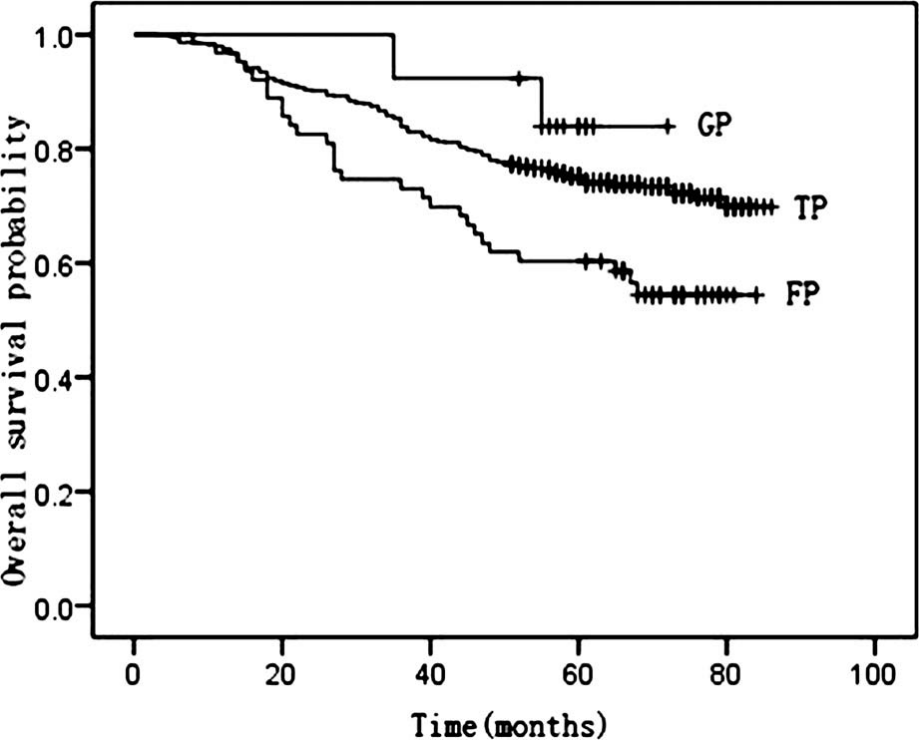
Kaplan-Meier curves of overall survival by TP, GP and FP regimens.

**Fig. 2:**
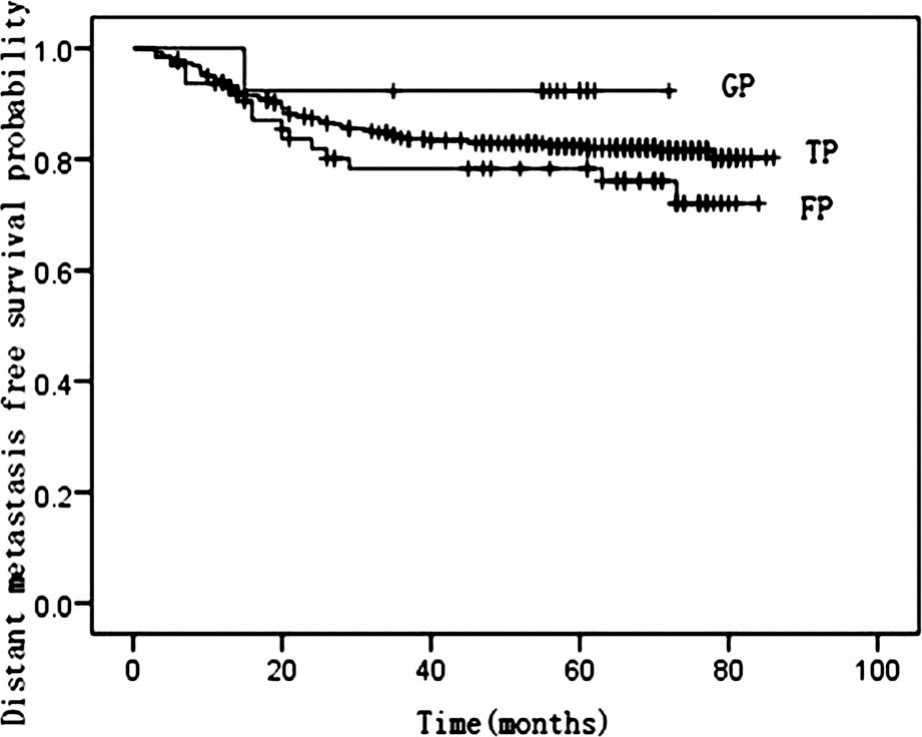
Kaplan-Meier curves of distant-metastasis free survival by TP, GP and FP regimens.

The associations between induction chemotherapy and OS/LRFS/DMFS rates are presented in Table-II. Induction chemotherapy regimens did not fit for LRFS log-rank test. Fig.1 and Fig.2 illustrate the association between OS and DMFS with different induction regimens (TP regimen; GP regimen; FP regimen).

**Table-II:** OS/ LRFS/ DMFS rates by TP regime, GP regime and FP regime.

Induction chemotherapy	OS rate			LRFS rate			DMFS rate		
	3-year (%)	5-year (%)	P	3-year (%)	5-year (%)	P*	3-year (%)	5-year (%)	P
TP	83.8	75.1		91.3	88.2		83.9	82.4	
GP	92.3	83.9	0.009	76.9	76.9	/	92.3	92.3	0.286
FP	73.0	60.3		85.2	76.9		78.3	78.3	

* Owning to the value, induction chemotherapy regimens did not fit for LRFS log-rank test.

### Prognostic factors

Various potential prognostic factors including gender, age, stage, Histology, radiotherapy technology, concurrent chemotherapy, induction chemotherapy, lymph node, retropharyngeal lymph nodes involved, lymph nodes extracapsular invasion, chemotherapy cycle, T/N-classification, induction chemotherapy regimens (TP; GP; FP), distant metastasis and relapse on predicting OS/LRFS/DMFS rates were evaluated in both univariate and multivariate analyses.

In preliminary univariate analysis, gender (P=0.006), age (P<0.001), stage (P<0.001), radiotherapy technology (P=0.042), lymph nodes extracapsular involved (P=0.001), metastasis (P<0.001), recurrence (P<0.001), induction chemotherapy regimens (P=0.009) were significant prognostic factors for OS. Stage (P=0.012) was a significant prognostic factor for LRFS. And gender (P=0.032), N-classification (P=0.007), stage (P<0.001), lymph node (P=0.005), retropharyngeal lymph nodes involved (P=0.006) and lymph nodes extracapsular invasion (P<0.001) influenced DMFS.

In multivariate analysis, induction chemotherapy was not a significant prognostic factor for OS (P=0.193), but multivariate subgroup analysis showed that GP regimen was an independent predictor compared with TP and FP regimen for OS (RR=0.201, P=0.038). Furthermore, there was a positive tendency of induction chemotherapy for DMFS (P=0.088), and TP regimen was the independent predictor for DMFS (RR=0.561, P=0.038) and a trend toward improved DMFS with GP regimen was also observed, though this was not statistically significant (RR =0.189; P= 0.109). In addition, radiotherapy technology (RR = 0.676, P=0.037) was associated with good prognosis for OS. Age (RR = 1.972, P=0.000), relapse (RR = 3.821, P=0.000) and distant metastasis (RR = 10.205, P=0.000) were negative prognostic factors for OS. Lymph nodes extracapsular invasion and T-classification were found to be the negative independent predictors for DMFS (RR = 1.526, P=0.002; RR = 1.723, P=0.050) (Table-III and Table-IV). There was no significant independent factor for LRFS in multivariate analysis.

**Table-III:** Multivariate analysis for OS.

Factor	Regression coefficient	P	RR	95% CI Exp	
				Lower	Upper
Age	0.679	0.000	1.972	1.438	2.704
Radiotherapy	-0.392	0.037	0.676	0.467	0.977
Induction chemotherapy		0.193			
TP	-0.159	0.508	0.853	0.533	1.366
GP	-1.604	0.038	0.201	0.044	0.914
FP	0.006	0.983	1.006	0.576	1.758
Relapse	1.340	0.000	3.821	2.662	5.484
Metastasis	2.323	0.000	10.205	7.366	14.138

**Table-IV:** Multivariate analysis for DMFS.

Factor	Regression coefficient	P	RR	95% CI Exp	
				Lower	Upper
T classification	0.423	0.002	1.526	1.162	2.006
Induction chemotherapy		0.088			
TP	-0.577	0.038	0.561	0.326	0.968
GP	-1.667	0.109	0.189	0.025	1.452
FP	-0.161	0.658	0.851	0.417	1.737
Extracapsular invasion	0.544	0.050	1.723	0.999	2.970

## DISCUSSION

In our study, the 3- and 5-year OS, LRFS, and DMFS rates indicated that distant metastasis remained the major factor for treatment failure. Multivariate analysis also confirming that distant metastasis was the adverse factor for OS. This result was similar with other trials.^21^,^22^ The better result of OS rate in our study compared with previous study^2^ can be attributed to IMRT and chemotherapy. Induction chemotherapy was not an independent prognostic factor for OS (P=0.193), but had a positive tendency of for DMFS (P=0.088). GP regimen was an independent predictor for OS (RR=0.201, P=0.038) and a trend toward improved DMFS was also observed, though this difference was not significant (RR =0.189; P= 0.109). However, TP regimen was only an independent predictor for DMFS in multivariate subgroup analysis (RR=0.561, P=0.038). Probably, GP regimen was superior in survival benefit to TP/FP regimen.

Based on the 0099 trial,^4^ concurrent chemoradiation with or without adjuvant chemotherapy was the current standard care for locoregionally advanced NPC. Baujat et al.^23^ also demonstrated a significant increase of concurrent chemotherapy for both OS (6% at 5 years) and PFS (10% at 5 years) rates. However, the acute toxicities stopped many patients from completing the whole therapy. In our study, 74.67% patients discontinued concurrent chemotherapy mainly due to intolerable toxicities.

IMRT enabled the delivery of higher radiation dose to the primary disease and neck metastases while sparing OARs. The local and regional controls were particularly encouraging after IMRT, exceeding 95% in some of previous reports.^3^,^24^,^25^ With such a high locoregional control rate, there was few space left to improve therapeutic effect though improving LRFS. Since concurrent chemotherapy was used mainly to increase locoregional control, there was no survival benefit when adding concurrent chemotherapy to IMRT.^1^,^26^ Therefore, the effect of concurrent chemotherapy on locoregionally advanced NPC was decreasing gradually. To solve the problem of distant metastasis, adding induction or adjuvant chemotherapy was promising.

In summary, adding induction chemotherapy had no survival benefit, but GP regimen was an independent predictor for OS and had a trend toward improved DMFS, while TP regimen was only found to be an independent predictor for DMFS. GP regimen may be more effective than TP/FP regimen for treating locoregionally advanced NPC.
